# Long-Term Tracking of Free-Swimming *Paramecium caudatum* in Viscous Media Using a Curved Sample Chamber

**DOI:** 10.3390/mi9010007

**Published:** 2017-12-28

**Authors:** Mohiuddin Khan Shourav, Jung Kyung Kim

**Affiliations:** 1 Department of Mechanical Engineering, Graduate School, Kookmin University, 77 Jeongneung-ro, Seongbuk-gu, Seoul 02707, Korea; khan@kookmin.ac.kr; 2 School of Mechanical Engineering, Kookmin University, 77 Jeongneung-ro, Seongbuk-gu, Seoul 02707, Korea

**Keywords:** curved chamber, particle tracking, field curvature, microswimmer, large field of view

## Abstract

It is technically difficult to acquire large-field images under the complexity and cost restrictions of a diagnostic and instant field research purpose. The goal of the introduced large-field imaging system is to achieve a tolerable resolution for detecting microscale particles or objects in the entire image field without the field-curvature effect, while maintaining a cost-effective procedure and simple design. To use a single commercial lens for imaging a large field, the design attempts to fabricate a curved microfluidic chamber. This imaging technique improves the field curvature and distortion at an acceptable level of particle detection. This study examines *Paramecium caudatum* microswimmers to track their motion dynamics in different viscous media with imaging techniques. In addition, the study found that the average speed for *P. caudatum* was 60 µm/s, with a standard deviation of ±12 µm/s from microscopic imaging of the original medium of the sample, which leads to a variation of 20% from the average measurement. In contrast, from large-field imaging, the average speeds of *P. caudatum* were 63 µm/s and 68 µm/s in the flat and curved chambers, respectively, with the same medium viscosity. Furthermore, the standard deviations that were observed were ±7 µm/s and ±4 µm/s and the variations from the average speed were calculated as 11% and 5.8% for the flat and curved chambers, respectively. The proposed methodology can be applied to measure the locomotion of the microswimmer at small scales with high precision.

## 1. Introduction

With considerable advancements in the development of microscopes, it is now possible to investigate particles and organisms at a microscopic scale, which is something that was never possible with the naked eye. These organisms and particles are currently being studied by scientists and researchers to characterize their behavior. However, the increasing demand of this technology in various research and application fields raises some issues that are worth considering. To observe spontaneous micro-organisms and measure particle behaviors in a velocity field, it is required to gain maximum dynamic range in a single image.

This study aims to introduce a relatively simple and inexpensive method of analyzing a large number of biological samples in a single microscopic image. Optical aberrations, particularly field curvature, should be eliminated in order to detect the signal at the periphery of the image, and the system must be simple so that image analysis can be performed with an image processing protocol. Some fast-moving biological organisms can be studied in a large microscopic imaging field to analyze their behavior as for drug discovery. Additionally, for precise measurement or counting purposes, a large field image can reduce the time and burden. Viscosity has a predominant influence on micro-organism’s flow motion, and the swimming speed of some bacteria and spermatozoa vary as the viscosity of the surrounding media changes [1].

Traditional microscopes can achieve high resolution over an entire image field. However, they possess a critical issue associated with the field of view (FOV), as they can only capture a small field. Benjamin et al. proposed adaptive scanning optical microscopy (ASOM) [2], and a unique wide-field imaging system has been designed with an inexpensive technique when compared with conventional microscopy. They introduced a microelectromechanical system deformable mirror to achieve a large-field imaging system, and their prototype could cover a 5-mm-wide FOV. However, it appears with a complex design of lens array. A high-throughput imaging system without using a lens has been proposed by Arpali et al. for a large volume of sample imaging [3], and a special microfluidic chamber has been designed in this regard, which it has good resolution over the image field. As the chamber is placed over the charge-coupled device (CCD) sensor, it does not show the aberrations in the imaging system that are caused by the regular lens. However, in this case, a large CCD sensor is used, requiring high optical expertise to design the system. There are also other approaches for obtaining large FOV imaging. Cho et al. demonstrated a dual-mode high-throughput microscopy (DM-HTM) system to detect a large area of asbestos samples [4]. The stages of this system move to collect the images at various areas of the samples. However, these samples are at a static position, and some living organisms or particles in a velocity field may have high-speed movement, which makes it difficult to monitor with small field or scan the sample. Instead of scanning, a large FOV imaging technique is used for monitoring the *Caenorhabditis elegana* response to different chemical substances [5]. However, a microfluidic chamber was fabricated and a considerably expensive electron microscope was used for that technique. Several approaches have been proposed to extend the FOV, including a micro-array-based microscope [6], wherein a number of microlenses were used to extend the image field and wide-field confocal macroscope [7]. However, these approaches are considerably useful for analyzing predominately static specimens over dynamic measurement.

This report describes a simple optical imaging system for the large-field monitoring of dynamic measurement. A technique is also described for correcting the field-curvature effect while using a single lens. Typically, single-lens imaging system has some aberrations in the image field, but the sample can be detected if it is corrected for field curvature. The curved substrate has been proposed in our previous research [8] for demonstrating its performance of fluorescent cell imaging in comparison with a flat substrate. The proposed system does not require further image processing and can analyze the sample with an acquired single wide FOV raw image. When considering the resource-limited area and mass production for helping science education among young people, this system is relatively inexpensive. A rapid analysis can be also performed with this system in various research fields, particularly in diagnosis.

## 2. Materials and Methods

### 2.1. Optical Imaging System 

**Figure 1:**
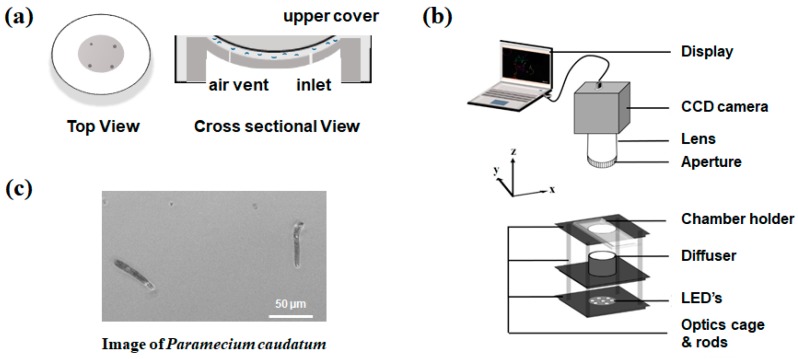
(**a**) Curved microfluidic chamber with a diameter of 12 mm; (**b**) A charge-coupled device (CCD) camera is connected to a computer to observe the real-time image sequence. The chamber holder, diffuser, and light source were caged together to hold still and illuminate the sample uniformly; and (**c**) *Paramecium caudatum* is used as the study sample, which has a size of 47.9 ± 5.5 μm.

In our optics setup with a bright-field imaging platform, microswimmers are located within a large-area curve substrate. A curved sample chamber is shown in Figure 1a. The proposed curved microfluidic chamber that is corrected for field curvature is implemented in this study. The curved chamber comprises two parts: a lower substrate and an upper window. It has a spherical dome shape in both parts, and this shape depends on the lens curvature, which was extensively described in our previous research [8]. The chamber that is used herein also has a 100 µm gap between the upper and lower parts and a 12-mm diameter. The flat chamber was further fabricated with identical dimensions to that of the curved chamber. Both the sample chambers are observed to have a closed system and the microswimmers cannot move out of the image frame. This is how the microswimmers are monitored. The work distance becomes large because of a large FOV target, making sample handling a convenient task. 

A commercial CCD camera sensor (DMK 51BU02, Imaging Source, Bremen, Germany) with 1.9-megapixel resolution and 4.4 µm pixel size was used. In addition, a biconvex lens (LB1014, Thorlabs, Newton, NJ, USA), having a focal length of 25 mm and a refractive index of 1.517, was used. The camera sensor was placed at the best focus distance and was connected to a computer via a USB cable, and images were taken using the IC Capture software package. A white LED light array was used for imaging the sample, as shown in the schematics of Figure 1b. Then, a diffuser (DG10-1500-MD, Thorlabs, Newton, NJ, USA) was used between the light source and the sample to uniformly distribute the light. 

A certain FOV was maintained with respect to optical magnification and resolution. The distance between the object and the lens was maintained at 70 mm to achieve an optical magnification of 0.54×. The image sensor was placed at the best focus distance from the backside of the lens, and a comparison was shown for the flat and curved substrates in the Results & Discussion section.

### 2.2. Sample Preparation

The average size of the microswimmers was 47.9 ± 5.5 µm, as shown in Figure 1c, which was measured in the expanded position of the microswimmers. *Paramecium caudatu* were then added with different concentration of methylcellulose (MC) and were centrifuged gently for a few minutes. The viscosity of the sample suspension and added MC were then measured using a commercially available viscometer (µVISC, H1310-00278, RheoSense, CA, USA), and the corresponding values are depicted in Table 1, which exhibits a reading resolution of 0.001 mPa·s. The accuracy of the reading is claimed to be up to 2% from the device company.

### 2.3. Image Acquisition

Images with a spatial resolution of 1600 × 1200 pixels were obtained using a homemade microscope system through a lens with a CCD camera (DMK 51BU02, IMAGEINGSOURCE, Bremen, Germany). Furthermore, the observed images were continuously captured at 13 fps for 2 min. Each image was preprocessed using the ImageJ 1.51i software package (National Institutes of Health (NIH), Bethesda, MD, USA). A blurred image of the first image in the stack was created and subtracted from the entire stack to reduce the background for conversion into a threshold stack [9]. To be more specific, the background was subtracted using the background subtraction process. Then, the threshold process was employed to highlight the microswimmers in the image. The movements of the microswimmers were tracked using a custom-programmed ImageJ’s multitrack (MTrack2) plugin (NIH, ImageJ version 1.51i; Nico Stuurman, University of California, San Francisco (UCSF)).

### 2.4. Measurement of Velocity Field and Trajectories

**Figure 2:**
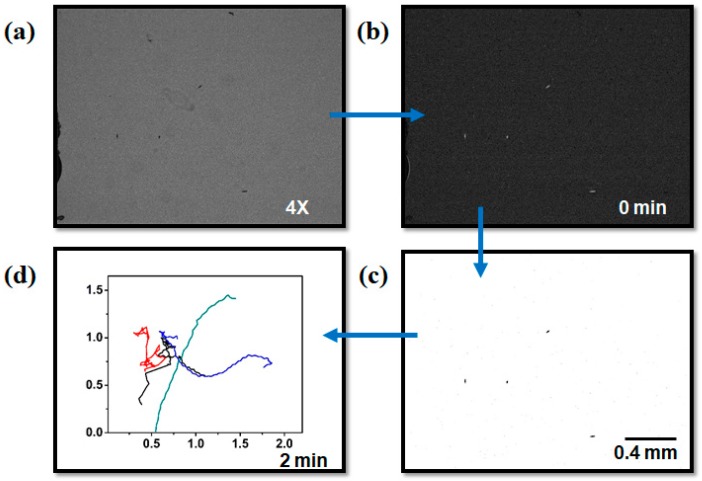
Image processing steps. (**a**) 4× magnified image of microswimmers at the original concentration; (**b**) Subtracted background; (**c**) The binarized image that separates the microswimmers from the background; and, (**d**) Microswimmer trajectory from a stack of images that were captured continuously for 2 min with an interval of 1 s. (Scale bar = 0.4 mm).

Two-dimensional (2D) microswimmer tracks were generated in a standard way using an automatic tracking method in ImageJ to approximate real diffusion and their behavior for the different viscous environments. The process for the interpretation of the image is shown in Figure 2, and we performed sampling of the microswimmer at a video-rate of 12 fps, which was the same as that for experimental imaging, with track durations of typically 1 s. The velocity of the microswimmer was calculated in a spreadsheet program by calculating the straight-line distance between centroid positions at an interval of 1 s, and 100-frame sequential images were tracked with the movement of individual microswimmers. The coordinates that were obtained from the trajectory were used to find the instantaneous speeds of the motion of microswimmers. The swimming speeds of the microswimmers that were affected by the viscous environment were analyzed by their trajectories. The movements of a microswimmer in each frame were averaged to obtain its speed, and different viscous environments were compared.

The path length was measured using the following equation:

(1) $$l=\sqrt{{\left(x_{2}-x_{1}\right)}^{2}+{\left(y_{2}-y_{1}\right)}^{2}}\;+\;\ldots \;+\;\sqrt{{\left(x_{n}-x_{n-1}\right)}^{2}+{\left(y_{n}-y_{n-1}\right)}^{2}}$$

The position changes of the microswimmers in the *x*- and *y*-directions were recorded for the whole image stack, and the distances traveled in each frame were summed for all of the images to measure the total traveled distances.

## 3. Results & Discussion

**Figure 3:**
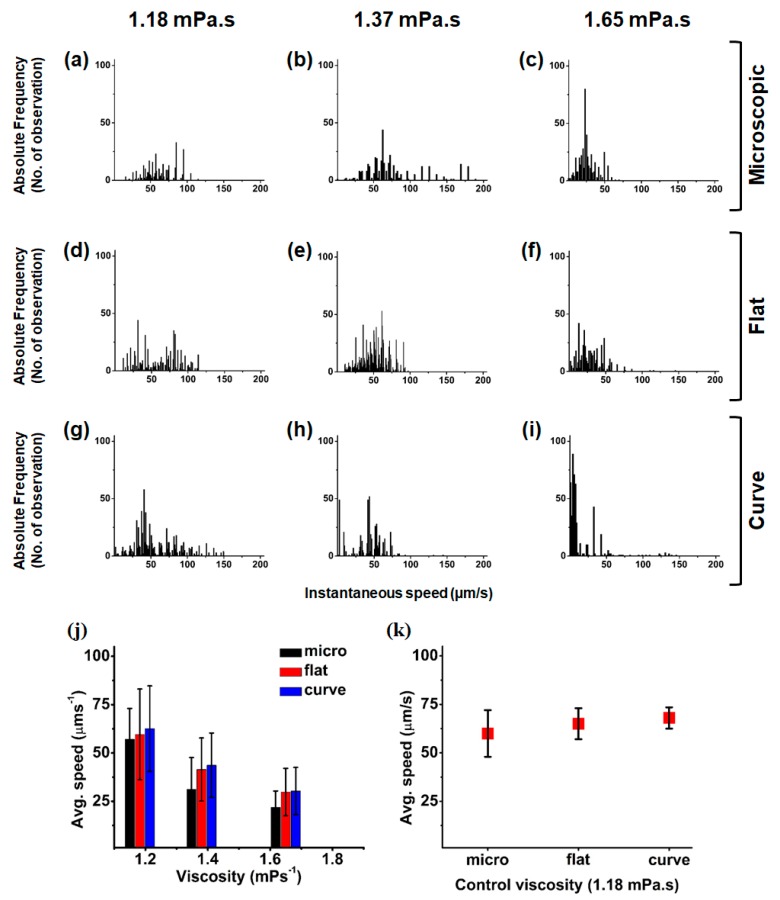
The instantaneous speeds of the microswimmers with histogram plots for the microscopic flat-chamber images (**a**–**c**); Large field-of-view (FOV) flat-chamber images (**d**–**f**); and large FOV curved-chamber images (**g**–**i**); The image sequences for 2 min were analyzed. The sample solution to which 0%, 5%, and 10% methylcellulose (MC) added is depicted in each column, respectively; (**j**) The average speeds of microswimmers in three different viscous media are illustrated in the plot. With increasing viscosity, the average speed decreases. All the three media exhibit the similar speed trends; (**k**) The plot depicts the average speeds of the microswimmers in the same viscous medium that is placed in three different sample chambers. In microscopic view, the deviation is observed to be larger than that in a large FOV image. The deviation is observed to be even smaller in the curved chamber, as compared to the flat chamber.

MC was added to the sample solution at ratios of 5% and 10%. Figure 3a–c show the instantaneous speed of the microswimmers under the commercially available 4× microscopic imaging system, where the field size is 2.2 mm × 1.76 mm. The speed was measured using three media of different viscosities; sample solution without MC, sample solution with 5% MC, and sample solution with 10% MC. 

The viscosities for those solutions are shown in the Material and Methods section. Figure 3d–i show the instantaneous speed of the microswimmers in flat and curved chambers, respectively, with the three different viscous media. With increasing viscosity, the average speed decreased and a similar average speed was observed for our test micro-organism: *P. minimum* (Dinophyceae). Sohn et al. [10] observed that the average speed of *P. minimum* (Dinophyceae) is approximately 51.26 µm/s at a viscosity of 1.12 mPa·s, whereas 56.9 µm/s was obtained for *P. caudatum*.

The images are taken for 2 min with an interval of 1 s, and the images show that the microswimmers did not maintain the same speed in each movement from the instantaneous speed measurement. However, the average speed is nearly the same in all of the imaging conditions. The results show that large-field imaging could yield more accurate measurements than the small field. In addition, the average speed of the microswimmers was 60 µm/s with a ±12 µm/s standard deviation, which leads to a 20% variation from the average measurement. In contrast, from large-field imaging, the speeds of the microswimmers in the flat and curved chambers were 63 µm/s and 68 µm/s, with standard deviations of ±7 µm/s and ±4 µm/s, respectively. The variations in the average speeds were also calculated as 11% and 5.8% for the flat and curved chambers, respectively. There are 10–15 microswimmers in the image field of microscopy with the instantaneous speeds that are illustrated in Figure 3. Further, the average speed was obtained using three sets of measurement. 

**Figure 4:**
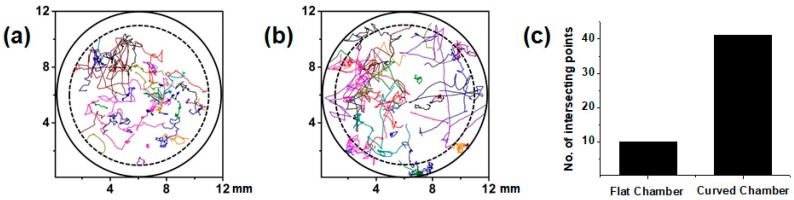
Trajectories from large-field flat and curved chamber image sequences. (**a**) The trajectories of the microswimmers moving in the flat chamber are trimmed at the edge of the image fields; (**b**) The trajectories of the microswimmers in the curved chamber are shown clearly even at the edge of the image field; and, (**c**) Comparison of the number of intersection points between the trajectories and inner circle of the 10 mm diameter dash line that is depicted in (**a**,**b**).

A quantitative analysis was performed in the flat and curved chambers using single-lens imaging. The flat chamber was imaged under microscopic and single lens imaging systems. Microscopic image analysis is considered to be a reference measurement. Figure 4 illustrates a comparative study of these two conditions. The microswimmer trajectories that were obtained using large-field flat and curved chamber image sequences are depicted in Figure 4a,b, respectively. Further, these trajectories were evaluated for the scenario where 0% MC was added to the media of the samples, and the large-field images had an area of 12 mm × 12 mm. 

Microswimmers are left free to move without any external influence. From their random motion, we can obtain the corresponding trajectories. Further, we observe that curved chamber trajectories tend to cover a complete track of 2 min. However, we observe that some of the trajectories terminate before complete their execution because of the field curvature effect at the edge of the chamber. However, it is not necessary to have a small tracking number frame or short distance in flat chamber as long as the microswimmers reach the periphery of the chamber. However, the average speed is observed to exhibit more deviation in the flat chamber than in the curved chamber due to the presence of some broken trajectories. The trajectories of the microswimmers in the flat and curved chambers were then compared. In addition, the trajectory of the microswimmers in the entire image field for the curved chamber was traced. Interestingly, for a flat chamber, the movements of the microswimmers were not observed at the periphery. Figure 4 shows the trajectory of the microswimmers in the flat and curved chambers. At the periphery, no microswimmers were observed because of the field-curvature effect. This optical aberration has a similarity with a petzval surface [11]. While this aberration can only be observed in a perfect lens when corrected for astigmatism [12], there are many groups of researchers who have worked on eliminating this aberration. Furthermore, an electronic eye was developed [13]. Using a curved image plane [14,15] was one of the approaches to eliminate this problem. This problem and a solution for static measurement have been demonstrated in our previous research [8] by developing curved microfluidic chambers.

## 4. Conclusions

As the average speed of microswimmers exhibited a smaller standard deviation in large FOV imaging, the variation in the speed measurement is less than that in small FOV analysis, resulting in higher precision. However, the curved chamber has an even smaller deviation than the flat chamber and further exhibits higher average speed due to long-term particle tracking. This could be developed as an inexpensive large FOV optical imaging system and be portable for the particles, microswimmers, and various dynamic measurements of samples. Furthermore, the results of this research will help in studying the velocity field analysis and the locomotion of other living micro-organisms. 

## Figures and Tables

**Table 1:** Viscosity measurement of the concentration of methylcellulose (MC) used in the experiment.

Viscous Liquid	Unit (mPa·s)
Control	1.182
Methylcellulose (MC) (5%)	1. 379
MC (10%)	1.65
MC (100%)	23.85
